# Deletion of Brg1 causes abnormal hair cell planer polarity, hair cell anchorage, and scar formation in mouse cochlea

**DOI:** 10.1038/srep27124

**Published:** 2016-06-03

**Authors:** Yecheng Jin, Naixia Ren, Shiwei Li, Xiaolong Fu, Xiaoyang Sun, Yuqin Men, Zhigang Xu, Jian Zhang, Yue Xie, Ming Xia, Jiangang Gao

**Affiliations:** 1 School of Life Science and Key Laboratory of the Ministry of Education for Experimental Teratology, Shandong University, Jinan 250100, China; 2 Department of Otolaryngology-Head and Neck Surgery, The Second Hospital of Shandong University, Jinan 250033, China

## Abstract

Hair cells (HCs) are mechanosensors that play crucial roles in perceiving sound, acceleration, and fluid motion. The precise architecture of the auditory epithelium and its repair after HC loss is indispensable to the function of organ of Corti (OC). In this study, we showed that Brg1 was highly expressed in auditory HCs. Specific deletion of Brg1 in postnatal HCs resulted in rapid HC degeneration and profound deafness in mice. Further experiments showed that cell-intrinsic polarity of HCs was abolished, docking of outer hair cells (OHCs) by Deiter’s cells (DCs) failed, and scar formation in the reticular lamina was deficient. We demonstrated that Brg1 ablation disrupted the Gαi/Insc/LGN and aPKC asymmetric distributions, without overt effects on the core planer cell polarity (PCP) pathway. We also demonstrated that Brg1-deficient HCs underwent apoptosis, and that leakage in the reticular lamina caused by deficient scar formation shifted the mode of OHC death from apoptosis to necrosis. Together, these data demonstrated a requirement for Brg1 activity in HC development and suggested a role for Brg1 in the proper cellular structure formation of HCs.

The mammalian auditory sensory epithelium, the OC of the cochlea, is composed of mechanosensory hair cells (HCs) that convert sound energy into electrical signals, which are in turn transmitted to the central nervous system. The hair bundle on the apical surfaces of HCs consists of rows of actin-based stereocilia with graded heights that form a V-shaped staircase pattern and act as the mechanotransduction organelle of the HCs. The actin-based hair bundles on HCs uniformly aligned on the apices of HCs with V-shapes pointing unidirectionally towards the outer (lateral) border of the cochlear duct. The HC planer polarity is essential for the correct perception of sound.

The HCs are interdigitated with supporting cells(SCs) in the OC, forming a checkerboard-like cellular pattern. The SCs include the inner phalangeal cells (IPhCs), the inner and outer pillar cells (IPCs and OPCs), and the Deiter’s cells. The SCs project phalangeal cellular processes toward the lumen of the cochlear duct. The apex of HCs form tight cellular contacts with the flattened ends of phalangeal processes of SCs through a special tight junction hybridized with the adherens junction^1^, and the OHC base is encapsulated by a cup-like subdomain that differentiates from the DC soma^2^. The tight junctions play an important role in compartmentalizing the endolymph and perilymph, the compositionally distinct inner ear fluids^3^,^4^. When HC death is triggered by noise, ototoxic drugs and the effects of aging, the epithelium must be repaired, which is called scar formation. Scar formation is complicated by the actin-rich cuticular plates of HCs and by the F-actin belts of DCs. Prompt sealing of the epithelial surface by scar formation prevents the expansion of damage by limiting the entry of the potassium-rich endolymph into the organ^5^,^6^,^7^. Although the structural integrity is essential to hearing, perhaps at the expense of its morphological complexity, HC regeneration does not occur in the mammalian OC^8^.

Brg1 is an ATPase subunit of the SWI/SNF complex that is involved in nucleosome mobilization during development and tumorigenesis. Studies based on genetic analysis of human tumors have indicated that BRG1 is a tumor suppressor^9^,^10^,^11^,^12^. Brg1 null mutant mice die during the peri-implantation stage in embryonic development, and Brg1 heterozygous mice are prone to forming subcutaneoustumors^13^. ChIP-seq analysis suggests that Brg1 can bind active and suppressive regulatory sequences in a cell type- and developmental stage-specific manner^14^. It was shown that Brg1 is essential for many developmental processes in different tissues^15^,^16^,^17^,^18^,^19^,^20^. Although Brg1 is important in many developmental processes of different cell types, its role in HC development has not been reported.

To investigate the roles of Brg1 in HCs, we conditionally deleted Brg1 in auditory HCs using the Cre/LoxP system. Here, we found that Brg1 played important roles in HC intrinsic polarity maintenance, anchoring OHC base to the DCs and scar formation of the auditory epithelium. Deletion of Brg1 resulted in rapid HC degeneration and profound deafness, and HC degeneration was caused by a combination of apoptosis and necrosis. We demonstrated that the Gαi/Insc/LGN and aPKC asymmetric distributions were abolished in Brg1-deficient HCs, while the core PCP pathway was normal. These observations suggest that Brg1 is indispensable in the unique complex architecture formation and repair of the OC.

## Results

### Conditional inactivation of Brg1 in auditory HCs

**Figure 1:**
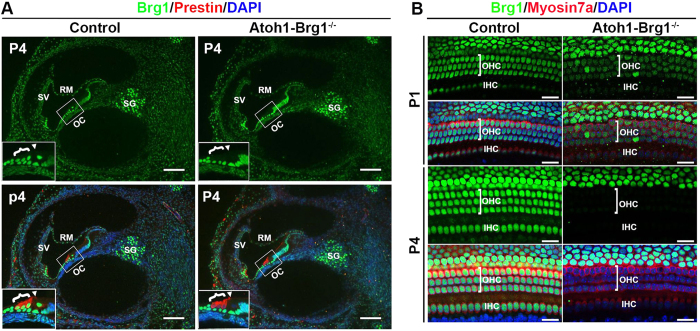
Brg1 expression in cochlea and conditional Brg1 inactivation in cochlear HCs. (**A**) Transverse sections of P4 cochlea stained with Brg1 (green), the OHC marker Prestin (red) and DAPI (blue, nuclei). Brg1 was specifically deleted in *Atoh1-Brg1*^−/−^ HCs. Inserts represent higher magnification views of the boxed areas. Brackets indicate OHCs, and arrowheads indicate IHC. Abbreviations: RM, Reissner’s membrane; SV, stria vascularis; OC, organ of Corti; SG, spiral ganglia. Scale bars: 100 μm. (**B**) Efficiency of Brg1 deletion shown by whole mount cochlea of P1 and P4 mice stained with Brg1 (green), the HC marker Myosin7a (red) and DAPI (blue, nuclei). Brg1 was sill detected in P1 *Atoh1-Brg1*^−/−^ HCs with a lower signal than in control HCs while in P4 *Atoh1-Brg1*^−/−^ HCs, Brg1 was absent in all HCs. Scale bars: 20 μm.

To determine Brg1 protein expression in the cochlea, we performed immunostaining using an anti-Brg1 antibody. As analyzed at E13.5, E15.5, E18.5, P4, P8, and p100, Brg1 was expressed in almost all cochlear cell nuclei and the signal was stronger in HCs, SCs, and ganglion than in other cell types (Fig. 1A; Supplementary Fig. S1). The expression of Brg1 indicated that Brg1 may play crucial roles in HCs.

To bypass embryonic lethality and investigate the function of Brg1 during HC development, we crossed *Brg1*^*flox*/*flox*^ mice with *Atoh1-Cre* mice that expressed Cre in developing HCs at approximately E14.5 to specifically inactivate Brg1 in HCs. The *Atoh1-Brg1*^−/−^ mice were viable and fertile and showed an indistinguishable gross morphology compared to control mice. At P1, Brg1 protein was still expressed in all HCs, although the signal was weaker than in controls (Fig. 1B). By P4, Brg1 protein was specifically deleted in all HCs (Fig. 1A,B). Delayed deletion of Brg1 protein compared to the expression of Cre recombinase may be due to the need for Cre recombinase to accumulate and the half-life of Brg1 protein.

### Brg1 inactivation led to profound deafness and severe cochlear HC degeneration

**Figure 2:**
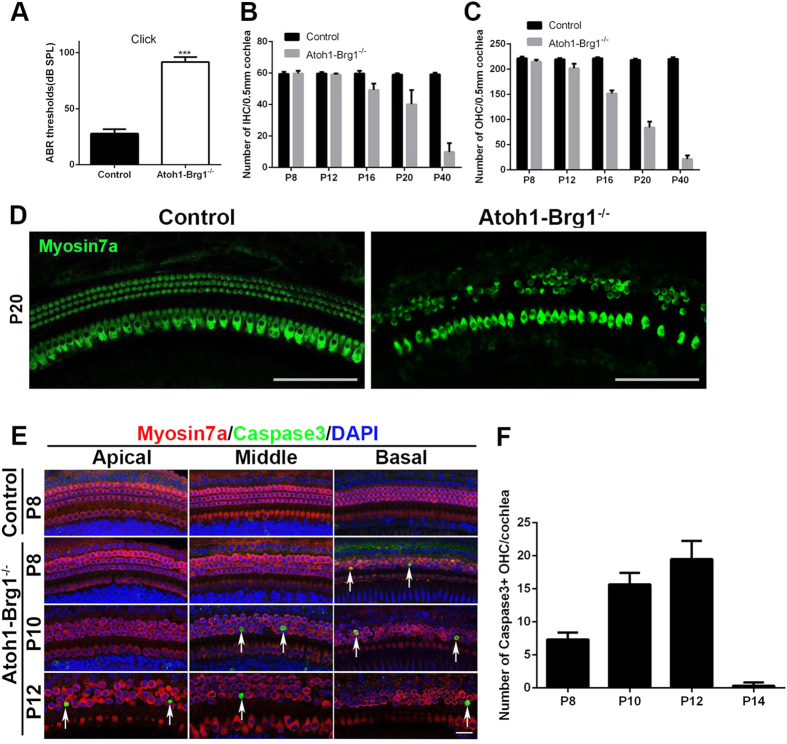
ABR analysis and HC loss in *Atoh1-Brg1*^−/−^ mice. (**A**) ABR measurements for broadband click of *Atoh1-Brg1*^−/−^ mice and wild-type mice at P20 (p = 3.5 × 10^−8^). The error bars indicate the SEM. ***p < 0.001 compared to the control by two-tailed Student’s t-test; n = 7 for controls and n = 9 for mutants. (**B**) Quantification of IHC number at the middle turn of the cochlea at different developmental stages. The error bars indicate the SEM. n = 3 animals for each group. (**C**) Quantification of the OHC number at middle turn of the cochlea at different developmental stages. The error bars indicate the SEM. n = 3 animals for each group. (**D**) Whole-mount images stained with the HC marker Myosin7a showing severe HCs loss at P20. Scale bars: 100 μm. (**E**) Whole-mount cochlea in different developmental stages stained with the apoptosis marker Cleaved-Caspase3 (green), the OHC marker Myosin7a (red) and DAPI (blue, nuclei). Arrows indicate apoptotic HCs labeled by Cleaved-Caspase3. Scale bars: 20 μm. (**F**) Quantification of the Cleaved-Caspase3-positive HC number of the *Atoh1-Brg1*^−/−^ cochlea at different developmental stages. The error bars indicate the SEM. n = 3 animals for each group.

To assess whether Brg1 inactivation in *Atoh1-Brg1*^−/−^ mice led to abnormal HC development, auditory brain stem responses (ABRs) were measured using a broadband click. At P20 and P40, the ABR thresholds in control mice were ~25 dB SPL (Fig. 2A; data not shown). Compared with control mice, the *Atoh1-Brg1*^−/−^ mice had significantly elevated ABR thresholds at P20 (~90 dB SPL) (Fig. 2A), and by P40, the ABR wave at 110 dB SPL (data not shown) could not be detected. Thus, the *Atoh1-Brg1*^−/−^ mice were profoundly deaf, indicating the important role of Brg1 in the postnatal development of HCs.

**Figure 3:**
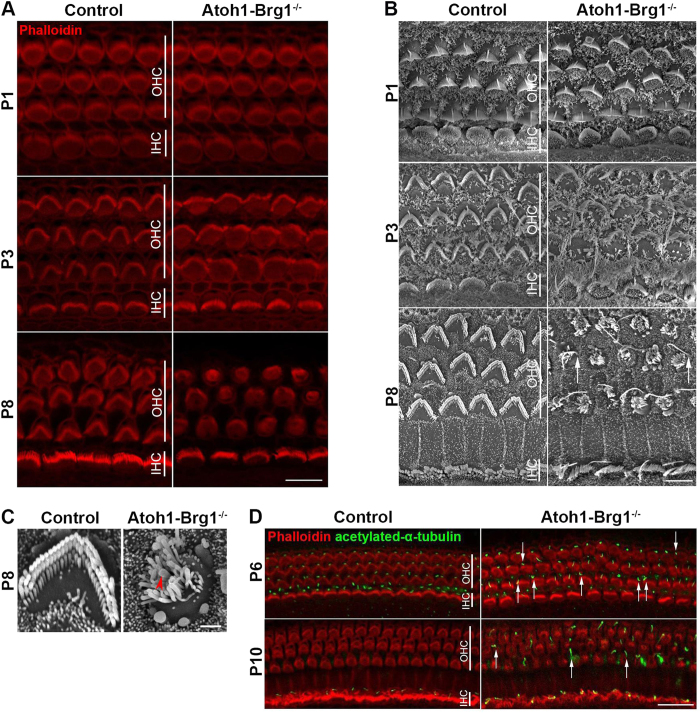
Apical morphology defect of *Atoh1-Brg1*^−/−^ HC. (**A**) Confocal images of HCs in the middle turn stained with phalloidin. Scale bar: 10 μm. (**B**) SEM images of stereocilia bundles in the middle turn of the cochlea. Note the long kinocilia in P8 *Atoh1-Brg1*^−/−^ HCs. Arrows indicate kinocilia that deviated from mediolateral axis. Scale bar: 5 μm. (**C**) Higher magnification SEM images of P8 stereocilia bundle in B. Arrowhead indicates a stereocilium that is much thicker than others. Scale bar: 1 μm. (**D**) Whole-mount cochlea of P6 and P10 mice stained with phalloidin(red) and the kinocilium marker acetylated-α-tubulin(green). Arrows indicate kinocilia deviated from the mediolateral axis. Scale bar: 20 μm.

To explore how Brg1 inactivation caused hearing loss, we dissected the cochlea of *Atoh1-Brg1*^−/−^ mice and found that the morphology of the temporal bones was not distinguishable between *Atoh1-Brg1*^−/−^ mice and control mice at a gross level (data not shown). Then, we examined whether HC loss occurred in the auditory epithelium. In the *Atoh1-Brg1*^−/−^ whole-mount specimen, we could not detect any HC loss before p8 (Figs 1b and 3D; data not shown). By P8, occasionally OHC loss was observed, mainly in the basal turn (Fig. 2B,C,E). By P12, OHC loss was observed along the length of the cochlea with a severity gradient from the base to apical turn, although the IHCs appeared largely intact (Fig. 2B,C,E). After P12, both the OHCs and IHCs underwent rapid cell death, and by P40, there were very few HCs in the cochlear epithelium (Fig. 2B,C,D). By 4 months of age, the spiral ganglion neurons were also reduced dramatically in *Atoh1-Brg1*^−/−^ mice resulting from HC loss, as observed in section by Hematoxylin and Eosin staining (Supplementary Fig. S2).

To confirm if Brg1-deficient HCs underwent apoptosis, we stained the whole-mount cochleae with an antibody against Cleaved-Caspase3, a marker of cells dying by apoptosis. In control HCs, we did not observe positive signals at any of the developmental stages tested (Fig. 2E; data not shown). In *Atoh1-Brg1*^−/−^ HCs, we did not observe a positive signal before P8 (data not shown). We observed several Cleaved-Caspase3-positive OHCs in the P8 *Atoh1-Brg1*^−/−^ cochlea, primarily in the basal turn (Fig. 2E,F). At P10, the signal of Cleaved-Caspase3 spread from the basal turn to the middle turn (Fig. 2E,F). At P12, the Cleaved-Caspase3-positive OHCs scattered along the length of the cochlea (Fig. 2E,F). The progress of Cleaved-Caspase3 signal spreading corresponded to the progress of HC loss. These results showed that Brg1-deficient OHCs underwent apoptosis after Brg1 deletion. Surprisingly, Cleaved-Caspase3 was rarely detected in P14 and older *Atoh1-Brg1*^−/−^ cochleae (Fig. 2F), showing that the mode of HC death changed after P14.

### Brg1 inactivation caused multiple HC apical morphology defects

To determine whether Brg1 deletion cause other cellular defect in addition to cell death, we examined the cellular morphology of HC in *Atoh1-Brg*^*−/−*^ mice. Confocal images and scanning electron microscopy (SEM) images revealed that the stereocilia bundles in *Atoh1-Brg1*^−/−^ mice were abnormal compared to those in control mice. At P1, the stereocilia bundle in *Atoh1-Brg1*^−/−^ cochlea and control cochlea had a comparable morphology (Fig. 3A,B), which was most likely attributed to the incomplete inactivation of Brg1 at the developmental stage (Fig. 1B). At P3, the arrangement of OHC stereocilia was somewhat disorganized and the V-shaped stereocilia bundles in OHCs exhibited a distinctly flattened shape in *Atoh1-Brg1*^−/−^ mice compared with wild-type littermates; some OHCs showed no obvious vertex for the V-shaped stereocilia bundles (Fig. 3A,B). At P8, most *Atoh1-Brg1*^−/−^ OHCs exhibited round-shaped hair bundles and V-shape stereocilia bundles existed in very few *Atoh1-Brg1*^−/−^ OHCs (Fig. 3A,B). Thicker stereocilia were observed in some *Atoh1-Brg1*^−/−^ OHCs (Fig. 3). However, there were no obvious differences between stereocilia bundles in *Atoh1-Brg1*^−/−^ and control IHCs (Fig. 3A,B). The abnormal stereocilia bundles showed that the cell-intrinsic polarity was deficient in Brg1-deficient HCs.[Fig f4][Fig f5][Fig f6]

Within the first postnatal week (P1–P7), control OHC apical circumferences/cuticular plates transited from rounded hexagons to a non-convex shape with two lateral lobes flanking a membrane concavity on the OHC medial side, and the hair bundle integrity was essential for the remodeling process^21^. Given that the hair bundles of *Atoh1-Brg1*^−/−^ OHCs were severely abnormal, we examined whether the remodeling of the apical circumferences/cuticular plates was affected. ZO-1 and βII-Spectrin staining showed that the apical circumferences/cuticular plates remained as rounded hexagons in *Atoh1-Brg1*^−/−^ mice at P8 (Fig. 7A; Supplementary Fig. S3A), which corresponded to round-shaped stereocilia bundles. This result showed that the remodeling of the apical circumference was deficient.

The kinocilium locates at the vertex of the V-shaped stereocilia bundle and retacts at about P10^22^. The organization of the stereocilia bundle and the location of the kinocilium have been used to measure the intrinsic polarity and orientation of each HC^23^. We observed that the kinocilia in some Brg1-deficient OHCs deviated from the mediolateral axis and were mispositioned relative to the bundle vertex (Fig. 3B,D). Additionally, the kinocilium in Brg1-deficient HCs was longer than in controls at P6 (Fig. 3D). By P10, we could not observe any kinocilium in control OHCs, but many *Atoh1-Brg1*^−/−^ OHCs had a long kinocilium on the apex (Fig. 3D). This result showed that the position and retraction of the kinocilium was abnormal and confirmed the cell-intrinsic polarity defect in *Atoh1-Brg1*^−/−^ HCs.

### The localization of the core PCP components Vangl1 and Fz6 were normal in *Atoh1-Brg1* ^−/−^ cochlea

**Figure 4 f4:**
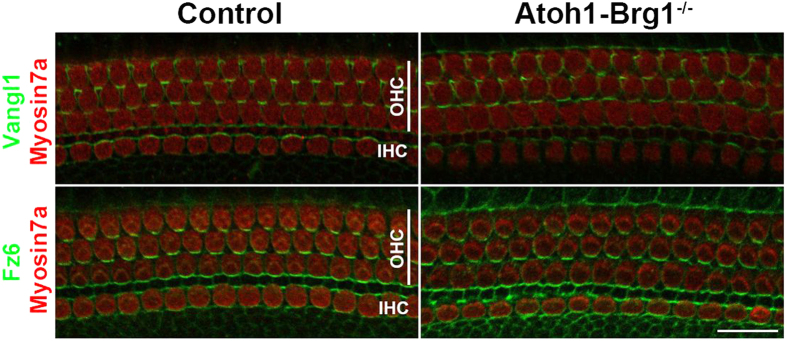
Localization of the core PCP components Vangl1 and Fz6 show no significant difference between control and *Atoh1-Brg1*^−/−^ cochlea. Upper panel shows whole-mount cochlea of P4 mice stained with Vangl1 (green) and the HC marker Myosin7a (red). Lower panel shows whole-mount cochlea of P4 mice stained with Fz6 (green) and the HC marker Myosin7a (red). Scale bar: 20 μm.

During tissue-level PCP establishment, core PCP proteins respond to upstream directional cues and locate asymmetrically at the cell membranes along the planar polarity axis. In mice, mutations in core PCP components cause the misorientation of the hair bundles and abnormal positioning of the kinocilium^24^,^25^,^26^,^27^. The kinocilia were mispositioned at the apex of some Brg1-deficient OHCs, a phenotype observed in core PCP mutant mice. To determine whether core PCP components were affected in the *Atoh1-Brg1*^−/−^ cochlea, we compared the distributions of Vangl1 and Fz6 in control and *Atoh1-Brg1*^−/−^ cochleae. Vangl1 and Fz6 immunofluorescence signals were concentrated along the boundary between the medial edges of HCs and the lateral edges of SCs in the control auditory epithelium (Fig. 4). The distributions of the signals for Vangl1 and Fz6 were almost the same in the *Atoh1-Brg1*^−/−^ auditory epithelium as in the control (Fig. 4). Together, these results suggested that core PCP signaling was still maintained in the *Atoh1-Brg1*^−/−^ cochlea.

### The Gαi/mInsc/LGN and aPKC asymmetric distributions and microtubule network at the apical surface was abnormal in Brg1-deficient OHCs

**Figure 5 f5:**
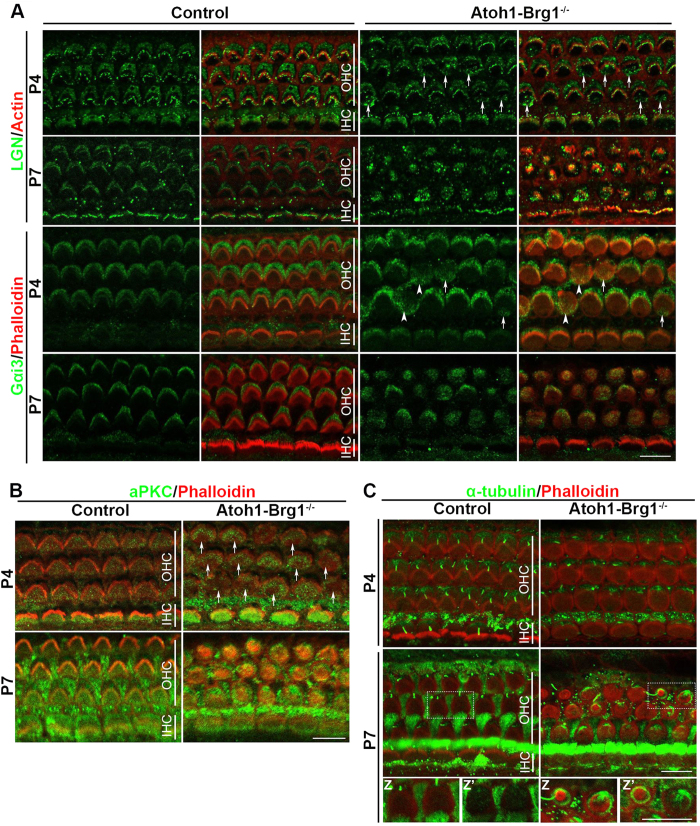
The Gαi/mInsc/LGN and aPKC asymmetric distribution, and microtubule network abnormality in Brg1-deficient OHCs. (**A**) The upper two panels show whole-mount cochlea of P4 and P7 mice stained with LGN (green) and Actin (red). The lower two panels show whole-mount cochlea of P4 and P7 mice stained with Gαi3 (green) and phalloidin (red). Arrows indicate OHCs with LGN/Gαi3 staining in the medial side adjacent to the medial edges of the apical surface in addition to lateral crescents. Arrowheads indicate OHCs with LGN/Gαi3 staining only in the medial side adjacent to the medial edges of the apical surface. Scale bar: 10 μm. (**B**) Whole-mount cochlea of P4 and P7 mice stained with aPKC (green) and phalloidin (red). Arrows indicate OHCs with aPKC-free areas near the medial membrane. Scale bar: 10 μm. (**C**) Whole-mount cochlea of P4 and P7 mice stained with α-tubulin (green) and phalloidin (red). Lowest panel shows higher magnification of dashed box optical taken at the level of the cuticular plate (Z) and 1 μm basal to Z (Z’). Scale bars: 10 μm.

It was reported that Gαi, mInsc, and LGN collectively exclude aPKC from the lateral region, leading to the compartmentalization of the HC apex, which acts as a blueprint to define the V-shaped contour of the stereocilia bundle and guide the relocalization of kinocilium^28^. We examined whether Gαi/mInsc/LGN and aPKC asymmetric distributions was abnormal in Brg1-deficient HCs. Given that Gαi, mInsc, and LGN interact with each other and form a complex at the HC apex to perform their function, we choose Gαi3 and LGN to represent the Gαi/mInsc/LGN complex. Gαi3 and LGN were asymmetrically segregated in all cochlear HCs, forming a crescent lateral to the stereocilia bundle at the apical surface in P4 and P7 control mice, while aPKC was asymmetrically localized in a cortical domain opposite and complementary to the Gαi3/LGN domain (Fig. 5A,B). The Gαi3 and LGN staining signals in IHCs were weaker at P7 than at P4, suggesting that Gαi3 and LGN may play smaller roles in the development of IHCs (Fig. 5A). In the P4 *Atoh1-Brg1*^−/−^ cochlea, abnormal Gαi3 and LGN crescents were still present lateral to the stereocilia at the apical surface of most OHCs. The crescents were flatter than those of the controls and corresponded to the flattened stereocilia bundles in the *Atoh1-Brg1*^−/−^ OHCs. In addition to the lateral crescent, in some *Atoh1-Brg1*^−/−^ OHCs, Gαi3 and LGN were also distributed on the medial side adjacent to the medial edges of the apical surface. We occasionally observed OHCs without Gαi3 and LGN lateral crescents; in these OHCs, Gαi3 and LGN staining were only detected in the medial half of the apical surface (Fig. 5A). Opposite and complementary to the abnormal Gαi3/LGN distribution, the aPKC expression pattern was altered in P4 Brg1-deficient OHCs. The expression pattern of aPKC was still on the medial side to the stereocilia on the majority of the OHC apical surface; however, there were aPKC-free areas of various sizes near the medial membrane in some Brg1-deficient OHCs compared to control OHCs (Fig. 5B). In P7 Brg1-deficient OHCs, Gαi3 and LGN showed irregular, round expression patterns of different sizes on most of the OHC apical surface, and the size largely matched the distribution of stereocilia (Fig. 5A). In the Brg1-deficient OHCs with irregular V-shaped stereocilia bundles, Gαi3 and LGN still showed an atypical crescent pattern and excluded aPKC at the lateral side (Fig. 5A,B). In Brg1-deficient OHCs with round-shaped stereocilia bundles, the aPKC distribution primarily surrounded the stereocilia bundles or spread all over the apical surface in some cases (Fig. 5B). These results demonstrated that Brg1 was essential for maintenance of the Gαi/mInsc/LGN and aPKC asymmetric distributions and confirmed the cell-intrinsic polarity defect in Brg1-deficient OHCs at the molecular level.

It was proven that Gαi, mInsc, LGN and aPKC recruit effectors, pulling on astral microtubules to position the mitotic spindle^29^, and were also important for the HC apical microtubule network^28^, so we examined whether the OHC microtubule network changed after Brg1 knockout. At p4, the microtubule distribution in Brg1-deficient OHCs showed some abnormality and was not distinctly different compared to that of control OHCs (Fig. 5C). In P7 control OHCs, the α-tubulin signal was much weaker than in P4 and more prominent lateral to the stereocilia of the cell (Fig. 5C). Consistent with abnormal Gαi/mInsc/LGN and aPKC asymmetric distributions in Brg1-deficient OHCs, the cytoplasmic microtubule pattern was dramatically altered at P7 (Fig. 5C). Obviously, the amount of microtubules was greatly increased in some Brg1-deficient OHCs than in controls (Fig. 5C). In OHCs with round-shaped stereocilia bundles, we observed strong α-tubulin stained microtubules that were organized into thick semi-circle or circular bundles that wrapped around the stereocilia bundle of the cell (Fig. 5C). We also observed disordered sub-bundles across the apical surface in some cells (Fig. 5C). The microtubule network of Brg1-deficient OHC was largely affected.

### OHC arrangement was disorganized, and OHC base was detached from DC in *Atoh1-Brg1* ^−/−^ cochlea

**Figure 6 f6:**
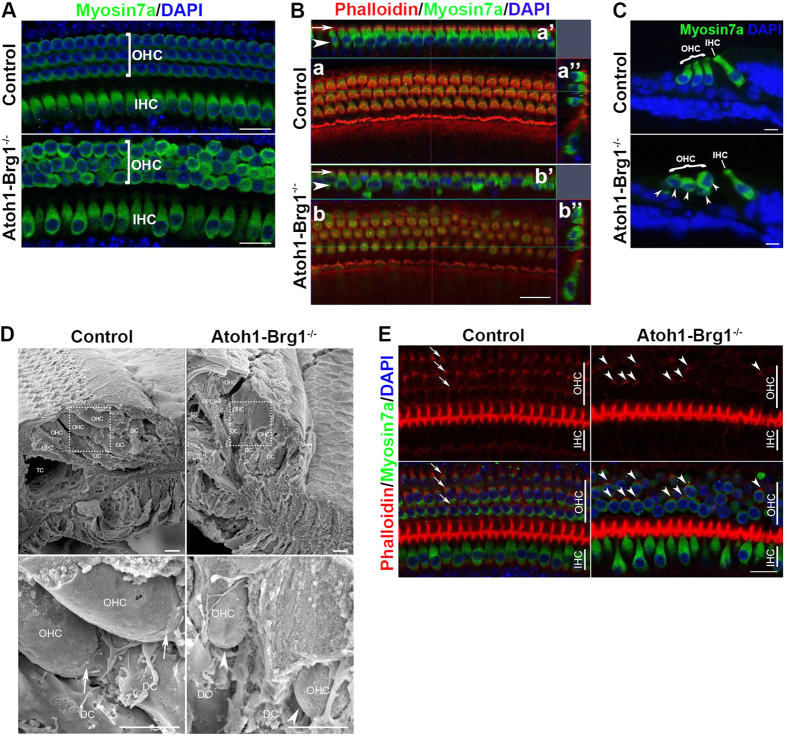
OHC arrangement was disorganized, and OHC base detached from DC in *Atoh1-Brg1*^−/−^ cochlea. (**A**) Whole-mount images of the middle turn in P8 cochlea stained with the HC marker Myosin7a (green) and DAPI (blue, nuclei). Scale bars: 20 μm. (**B**) Whole-mount cochlea of P8 mice stained with phalloidin (red), the OHC marker Myosin7a (green) and DAPI (blue, nuclei). Confocal Z-stack projections of reticular lamina show that the apical domain of HCs of *Atoh1-Brg1*^−/−^ mice is in a plane (a,b). Side views of reconstructed confocal images from a and b (a’,a”,b’,b”). Arrows indicate the apex of HCs, and arrowheads indicate the base of HCs. Scale bar: 20 μm. (**C**) Transverse sections of P10 cochlea stained with the HC marker Myosin7a (green) and DAPI (blue, nuclei). Arrow indicates disorganized arrangement of OHC base. Scale bars: 10 μm. (**D**) SEM images of OC in the middle turn of the cochlea. The lower panel represents a higher magnification of the dashed box. Arrows indicate DC cups in control mice, and arrowheads indicate a hanging OHC base in *Atoh1-Brg1*^−/−^ mice. Abbreviations: TC, tunnel of Corti; OPC, outer pillar cell; DC, Deiter’s cell. Scale bars: 5 μm. (**E**) Whole-mount cochlea of P10 mice stained with phalloidin (red), the OHC marker Myosin7a (green) and DAPI (blue, nuclei). Confocal Z-stack projections of the HC base showed that most of the DC cup actin plaque disappeared in *Atoh1-Brg1*^−/−^ mice. Arrows indicate three rows of actin plaques of DC cups in control mice, and arrowheads indicate that only a small subset of DC cup actin plaques exist in *Atoh1-Brg1*^−/−^ mice. Scale bar: 20 μm.

In the whole-mount cochlea images stained by HC marker Myosin7a, we observed that the OHC arrangement was disorganized in *Atoh1-Brg1*^−/−^ mice, while the IHC arrangement was almost not affected (Fig. 6A). Mild arrangement disorganization was first observed at P5, and by p8, the disorganization was obvious (Fig. 6A; data not shown). Confocal Z-stack projections of the reticular lamina showed that the apical surface of OHCs arranged in three regular rows, although the apical structure was abnormal (Fig. 6B). 3D reconstruction and section images further revealed that the OHCs were different in length and usually shorter than OHCs in control mice (Fig. 6B,C).

Based on the fact that Brg1-deficient OHC primarily showed a disorganized arrangement in the base, we hypothesized that the anchorage of the OHC base by DCs was deficient. The OHC base is docked by a cup-like subdomain (DC cup) that differentiates from the DC soma^2^. We did not observe the DC cup in either controls or *Atoh1-Brg1*^−/−^ OC at P6 (data not shown). In P8 control SEM images, we saw that a cup that was formed by DC encapsulated the OHC base (Fig. 6D). This showed that the DC cup formed between P6 and P8. In the P8 *Atoh1-Brg1*^−/−^ SEM images, we did not observe the DC cup. The OHC base was not docked by DC and was in a hanging state (Fig. 6D). Confocal microscopy images of the basal domain showed actin plaques of DC cups under every OHC in the control whole-mount specimen at P10 (Fig. 6E). In the *Atoh1-Brg1*^−/−^ specimen, most of the actin plaques disappeared (Fig. 6E). These results confirmed that Brg1 inactivation caused anchoring failure of the OHC base by DCs.

### Scar formation failed, and HC underwent necrosis after P14 in *Atoh1-Brg1* ^−/−^ cochlea

**Figure 7:**
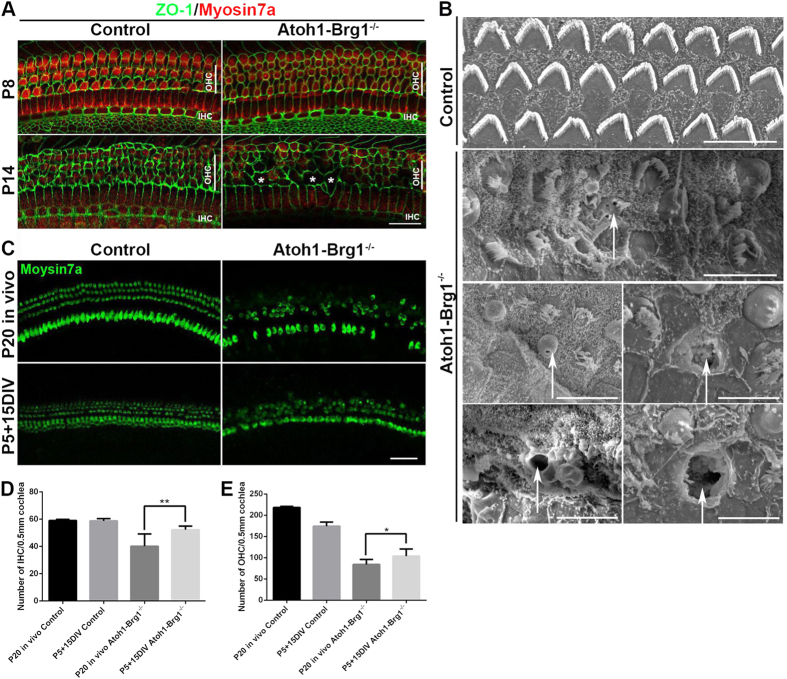
Broken reticular lamina after HC loss in *Atoh1-Brg1*^−/−^ mice and HC loss rescue *in vitro*. (**A**) Whole-mount cochlea stained with ZO-1 (green) and the HC marker Myosin7a (red). ZO-1 shown the same expression pattern in the control and *Atoh1-Brg1*^−/−^ auditory epithelia at P8. In P14 auditory epithelia, large areas without ZO-1 appeared at some sites where HC loss occurred. Asterisks indicate large ZO-1 free areas. Scale bar: 20 μm. (**B**) SEM images showing the holes in the P14 *Atoh1-Brg1*^−/−^ reticular lamina formed by the collapsed HC cuticular plate. Arrows indicate holes in the reticular lamina. Scale bars: 5 μm. (**C**) Whole-mount cochlea of explants of *Atoh1-Brg1*^−/−^ and control mice and of *Atoh1-Brg1*^−/−^ and control mice *in vivo* stained with the HC marker Myosin7a. Upper panel shows the cochlea specimen from P20 mice. The lower panel shows cochlea explants from the middle turn of the cochlea maintained in normal culture conditions from P5 to P20. Almost all of the IHCs survive in *Atoh1-Brg1*^−/−^ cochlea explants, and more OHCs survive compared with the *Atoh1-Brg1*^−/−^ cochlea from P20 mice *in vivo*. Scale bar: 50 μm. (**D**) Quantification of IHC number in the cochlea of explants of *Atoh1-Brg1*^−/−^ and control mice and of *Atoh1-Brg1*^−/−^ and control mice (p = 0.0089). The error bars indicate the SEM. **P < 0.01 compared to the control by two-tailed Student’s t-test; n = 6 cochleae for each group. (**E**) Quantification of the OHC number in the cochlea of explants of *Atoh1-Brg1*^−/−^ and control mice and of *Atoh1-Brg1*^−/−^ and control mice (p = 0.0396). The error bars indicate the SEM. *P < 0.05 compared to the control by two-tailed Student’s t-test; n = 6 cochleae for each group. DIV: day *in vitro*.

The apoptosis signal only existed between P8 and P12 in *Atoh1-Brg1*^−/−^ OHCs, but HC death was rapid after P14, including the IHCs. How did the HCs die after P14? The integrity of the reticular lamina is essential to limiting the entry of the potassium-rich endolymph into the OC, and leakage of the reticular lamina results in the entry of cytotoxic endolymph into the epithelium and eventually cell death by necrosis^2^,^30^. When HCs degenerate, the apices of SCs expand and occupy the area that was occupied by the lost HCs and form new tight junctions between the SCs, termed scar formation. Because the OHC apex had multiple deficiencies, we tested whether the tight junction was normal in the *Atoh1-Brg1*^−/−^ epithelium. Staining of the ZO-1, E-Cadherin, and β-Catenin, components of the special tight junction of the auditory epithelium^1^, showed that the tight junction in the *Atoh1-Brg1*^−/−^ epithelium was integrated (Fig. 7A; Supplementary Fig. S4A). The transmission electron microscopy (TEM) images also confirmed this result (Supplementary Fig. S4B). However, when OHC loss was obvious in *Atoh1-Brg1*^−/−^ cochlea by P14 (Fig. 2B,C), ZO-1 was absent at some sites that were previously occupied by HCs, forming a large ZO-1-free area (Fig. 7A). The ZO-1 staining pattern was located around the apical circumferences of HCs and SCs; therefore, we did not know whether the large ZO-1-free areas were larger expanding SCs or cell-free zones. Then, the SEM images confirmed the presence of some “holes” in the epithelium (Fig. 7B). The holes were formed by collapsed HC cuticular plates or loss of whole HCs (Fig. 7B). These results showed that scar formation was abnormal, and integrity of the reticular lamina was broken after HC loss in *Atoh1-Brg1*^−/−^ OC. Moreover, apoptotic HC death disappearance and accelerated HC death occurred after P14 (Fig. 2B,C,F), the same period when the ionic environment in the endolymph and high resting endocochlear potential (EP) appeared^31^. Thus, we drew preliminary conclusions that HC death after P14 in *Atoh1-Brg1*^−/−^ cochlea was a secondary effect mainly caused by the leakage of reticular lamina.

To determine whether accelerated degeneration of HCs in *Atoh1-Brg1*^−/−^ cochlea after P14 was triggered by leaked extracellular potassium-rich endolymph, we cultured explants derived from the OC of *Atoh1-Brg1*^−/−^ and control mice in DMEM culture medium containing 5.3 mM of K^+^ for up to 15 days (fromP5 to P20). In contrast to the *in vivo* P20 OC, many more HCs survived when OCs from *Atoh1-Brg1*^−/−^ mice were cultured *in vitro* (Fig. 7C,D,E). IHC degeneration in *Atoh1-Brg1*^−/−^ OC was largely rescued in explants, while the OHCs also showed less cell death than *in vivo* (Fig. 7C,D,E). Some OHC loss was also observed in control explants (Fig. 7C,E), which may be due to insufficient culture conditions for the long-term survival of OHCs. These data were consistent with the notion that the accelerated degeneration of HCs after P14 in the *Atoh1-Brg1*^−/−^ mice was due to unfavorable extracellular conditions caused by failed scar formation.

## Discussion

Previous studies have shown that Brg1 plays diverse roles in many different types of tissue, but its role in HC development remains unknown. We showed that Brg1 is highly expressed in the HCs of the inner ear in mice. Due to the embryonic lethality of the Brg1 null mutation, we specifically deleted Brg1 in HC to study its function. Conditional inactivation of Brg1 in the postnatal developing mouse HC causes rapid HC death and profound deafness. HC death was caused by a combination of apoptosis and necrosis in *Atoh1-Brg1*^−/−^ cochlea. We discovered that Brg1 in HCs was required for cell-intrinsic polarity maintenance, anchorage of the OHC base by the DC cup and scar formation of the auditory epithelium in postnatal development. The fact that HC intrinsic polarity establishment and scar formation are based on F-actin and that a F-actin plaque is also present in the DC cup cytoplasm, suggest that Brg1 may play a crucial role in coordinating multiple processes relative to F-actin. Nevertheless, the underlying mechanisms of these phenotypes caused by Brg1 inactivation remain unknown. Brg1 is reported to bind active and suppressive regulatory sequences of thousands of genes in a cell type- and developmental stage-specific manner^14^; hence, it is difficult to distinguish the primary mechanisms from secondary mechanisms involved in the developmental defects of *Atoh1-Brg1*^−/−^ HCs.

### Brg1 inactivation abolished the cell-intrinsic polarity of OHC

HCs display two levels of planer polarity. First, HCs are uniformly oriented in the cochlea, and the vertex of the V-shaped hair bundle on every HC points toward the lateral edge of the cochlear duct. This concerted tissue orientation is referred to as tissue-level PCP^32^. The core PCP pathway is required for the establishment of tissue-level PCP in the OC, as evidenced by random hair bundle orientation in mutant mice in which the core PCP components are inactivated^24^,^25^,^26^. Second, the V-shaped hair bundle morphology has a kinocilium located at the vertex of a single HC apex, and is termed cell-intrinsic polarity^33^. Few data on the mechanisms underlying the establishment of cell-intrinsic polarity are available. However, tissue-level PCP mutant HCs with misoriented stereocilia bundles usually have a largely intact apical morphology, suggesting that tissue-level and cell-intrinsic polarity pathway are largely independent from each other in planer polarity operations.

We demonstrated that the cell-intrinsic polarity was severely abnormal in Brg1-deficient OHCs, whereas the core PCP polarity pathway was largely intact in *Atoh1-Brg1*^−/−^ cochlea. The stereocilia bundle showed morphology defect at P2, and became round-shape in some OHC at P6 in *Atoh1-Brg1*^−/−^ cochlea (Supplementary Fig. S5). HC death in *Atoh1-Brg1*^−/−^ cochlea was first observed at P8. These results showed that stereocilia bundle morphology defect occurred prior to HC death. Additionally, even in the dying HCs, the V-shaped stereocilia bundle still exist^2^,^34^,^35^,^36^. Therefore, the cell-intrinsic polarity defect in *Atoh1-Brg1*^−/−^ HCs was not the secondary effect of cell death. The kinocilium is considered to be a lever that guides the orientation of the bundle inside the HC, and its centrifugal shift is required to establish a cell-intrinsic polarity. Although impairing ciliogenesis induces an aberrant stereocilia bundle in HCs, polarized stereocilia bundles still arise in most HCs^37^,^38^,^39^. Therefore, the cell-intrinsic polarity defect in *Atoh1-Brg1*^−/−^ HCs cannot be attributed to an aberrant kinocilium. Conditional mutants of Rac1 or Cdc42, members of the Rho family GTPases, also resulted in misoriented and misshapen stereocilia bundles^22^,^33^. In contrast, the cell-intrinsic polarity defect in Brg1-deficient OHCs was more severe, which showed an almost complete loss of bundle asymmetry. Thus, we ruled out the possibility that the cell-intrinsic polarity deficiency was caused by deficient Rac1 or Cdc42. Recent studies showed that Gαi, mInsc, and LGN collectively exclude aPKC from the lateral microvilli-free region, acting as a molecular blueprint at the apical surface of HCs to shape the bundle and cytoskeleton at the HC apex. Gαi is additionally required for migration direction of the kinocilium to couple the asymmetric stereocilia bundles with the core PCP pathway^28^,^40^. In the present study, Gαi3, LGN, and aPKC staining showed that the molecular blueprint complementary asymmetry was severely abolished and that the microtubule cytoskeleton network was largely abnormal in Brg1-deficient OHCs. The LGN and Gαi3 staining pattern corresponded to each other in both P4 and P7 Brg1-deficient OHCs; this result demonstrated that Brg1 deletion did not disrupt the interaction between LGN and Gαi3. Under forced expression of Gαi2 or LGN in the whole apex *in vitro*, the stereocilia bundle is constrained and forms a round-shape instead of a V-shape^28^, as in Brg1-deficient OHCs. Taken together, we concluded the abnormal Gαi/mInsc/LGN and aPKC complementary asymmetry caused by Brg1 deletion was responsible for the stereocilia bundle phenotype and kinocilium misorientation. Functional inhibition of Gαi using high doses of pertussis toxin *in vitro* results in round-shaped stereocilia bundles and a longer kinocilium, similar to that in Brg1-deficient HCs^40^. Thus, the longer kinocilium in Brg1-deficient HCs may be caused by aberrant Gαi function resulting from abnormal distribution. We noted the LGN and Gαi3 staining signals were much weaker in IHCs compared to OHCs, especially by P7. This suggested that the Gαi/mInsc/LGN complex may play a small role in the postnatal development of IHCs and could explain why the IHC stereocilia bundle was relatively normal.

Gαi/mInsc/LGN and aPKC distributions serve as a molecular blueprint to shape the hair bundle and cytoskeleton at the HC apex^28^. The distribution alteration of Gαi/mInsc/LGN and aPKC might occur in all mutant mice without normal V-shaped hair bundles. This hypothesis can be proven by analyzing the Gαi/mInsc/LGN and aPKC distributions in other mutants, such as *whirlin*^41^ and *clarin1*^42^ Usher mutants, in which the hair bundle morphology is abnormal. The underlying machinery of establishing Gαi/mInsc/LGN and aPKC distributions is poorly understood; thus, it is difficult to elucidate the cellular mechanisms of the broken apical molecular blueprint that caused by Brg1 inactivation. One possibility is that a Brg1 deletion disrupts the apical cytoskeleton that is essential for Gαi/mInsc/LGN anchorage, or abolishes one or more proteins involved in the anchorage of Gαi/mInsc/LGN to the apical cytoskeleton. Further experiments are required to study the mechanism of Brg1 in HC intrinsic polarity maintenance.

To our knowledge, the role of Brg1 in polarity has not been previously reported. When we were preparing the manuscript, Aldiri *et al*. published the mouse model for a Brg1 conditional deletion in the retina, which shows that Brg1 is essential for the polarity of the retina^43^. In their paper, aPKC and Par3 localization are deficient in the Brg1 depleted retina. In our study, aPKC localization was also abnormal in Brg1-deficient OHCs. Par3 is shown to co-localize with Gαi/mInsc/LGN in the embryonic auditory HC apex^28^. We detected the localization of Par3 in Brg1-deficient HCs in P4 and P6 mice. However, Par3 was only expressed in the apical junction of the auditory epithelium in control and mutant mice (Supplementary Fig. S6), which was the same result reported by Fukuda *et al*.^44^. This suggested that Par3 was downregulated in the postnatal HC apical surface and that Par3 may be not essential for the maintenance of the V-shaped stereocilia bundle in postnatal development.

### Brg1 loss caused failed anchorage of OHC base by DCs, and might induce anoikis

Brg1-deficient OHC disorganized arrangement was similar to the phenotypes in some mutant mice in which HCs reenter the cell cycle^45^,^46^. We also noticed that *Atoh1-Brg1*^−/−^ mice had larger OHC nuclei than control mice (Supplementary Fig. S3B,C). Brg1 was reported to be a tumor suppressor, and some research has shown that Brg1 plays important roles in Rb-mediated cell-cycle arrest^47^,^48^. Therefore, we asked whether Brg1-deficient OHCs reenter the cell cycle. We used antibodies against cell cycle markers PCNA, Ki67, Cyclin D1, and PH3 to label whole-mount cochlea. No positive signal was detected in either Brg1-deficient HCs or control HCs (Supplementary Fig. S7A,B; data not shown). BrdU incorporation experiments also showed no positive signal in Brg1-deficient HCs (Supplementary Fig. S7C). We excluded the possibility that Brg1-deficient HCs reenter the cell cycle. The nuclear morphology phenotype in Brg1-deficient OHCs may be due to the role of Brg1 in nuclear cytoskeleton regulation^49^.

The abnormal OHC shape and nuclear arrangement in *Atoh1-Brg1*^−/−^ cochlea was most probably caused by a failed anchorage of the OHC base. Although HC shrinkage is also induced by cell death^2^, the fact that cell arrangement disorganization occurs as early as P5 and that shorter OHCs were obvious at P6 in *Atoh1-Brg1*^−/−^ cochlea, earlier than HC death, ruled out the possibility that a HC morphology defect was caused by HC death. The cup of DCs is a poorly characterized cellular domain, and nerve terminals synapsing on the OHCs are also clustered inside the cup. How the DC cup forms, how the DC cup anchors HCs and what roles the DC cup plays in addition to anchoring OHCs is not well known. Moreover, the DC cup degenerates after HC death^2^.

Epithelial cells that have lost their cell-matrix or cell-cell anchorage will trigger cell suicide programs; this detachment-induced apoptosis is called anoikis^50^,^51^,^52^. We observed that the OHC base detached from DCs and that most DC cups disappeared in *Atoh1-Brg1*^−/−^ cochlea, a phenotype that has rarely been reported. The OHC disorganized arrangement was observed at P5 (data not shown), and by P8, the detachment was obvious in SEM images. Following detachment, the OHC underwent apoptosis. The time of apoptosis onset (~P8) was rapid after OHC detachment, but much later than Brg1 deletion and apical defect onset (~P2). In the current study, Brg1-deficient IHC death was mainly caused by unfavorable extracellular conditions after P14, and Brg1-deficient vestibular HCs in *Atoh1-Brg1*^−/−^ mice normally survived until P120 (data not shown). Given that Brg1-deficient IHCs and vestibular HCs, the other HC type in the inner ear, did not undergo apoptotic cell death suggests that Brg1 itself may not be essential for HC survival. Taken together, we propose that the OHC apoptosis in *Atoh1-Brg1*^−/−^ cochlea was anoikis. However, we cannot exclude the possibility that Brg1 plays different roles in OHC survival compared to IHCs and vestibular HCs, and further evidence is required to prove this hypothesis.

Many studies focus on the apical structure of HCs and overlook the cell junction between the HC base and the DC cup. The correct anchorage of the OHC base by the DC cup may be essential for OHC survival. Knowledge of how the OHC base anchors to the DC cup may be instrumental for stimulating regeneration and hearing therapy. The *Atoh1-Brg1*^−/−^ mouse line can be used as an animal model to explore the intercellular junction between the OHC base and the DC cup.

### Brg1 deletion in HCs led to the failure of scar formation after HC death

Apical surface closure by scar formation occurs after HC loss to preserve the intact reticular lamina and prevent leakage. After HC loss, the apices of SCs expand and rapidly occupy the apical surface in the sites of the lost HC. Studies based on the simpler epithelium show that the maintenance of the protective surface barrier depends on the actomyosin contractile mechanisms of adjacent cells and, in some cases, on the force originating from the apoptotic cell itself^53^,^54^,^55^,^56^. Scar formation in the auditory epithelium is based on F-actin remodeling, and during this process, DC extensions acquired new F-actin belts and the original belts were partially disassembled^2^,^6^,^57^. However, the underlying mechanism is largely unknown. We showed that the apical tight junction was integrated in *Atoh1-Brg1*^−/−^ OCs, but that “holes” appeared after HC loss in the auditory epithelium, suggesting that scar formation is disturbed. This result demonstrated that abnormal HCs can lead to the failure of scar formation. Deletion of Cdc42 in the cochlear SCs of postnatal mice also leads to deficient scar formation due to abnormal SC structural maturation^58^. In sum, these results demonstrate that the dying HC itself is essential in scar formation and that the expansion of the SC apex during scar formation requires proper collaboration between HCs and SCs. However, the apical structure of Brg1-deficient HCs is severely abnormal; we cannot conclude whether failed scar formation is caused by Brg1 inactivation per se or is a secondary effect of an abnormal HC structure.

## Materials and Methods

### Mice

All animal experimental procedures were approved by the Ethics Committee of Shandong University. Animal management was performed strictly in accordance with the standards of the Animal Ethics Committee of Shandong University (Permit Number: ECAESDUSM 20123004). *Brg1*^*flox*/*flox *^59 and *Atoh1-Cre*^60^ mouse lines were maintained on a mixed genetic background and genotyped as described previously. *Brg1*^*flox*/*flox*^ females were mated with *Atoh1-Brg1*^*flox*/+^ to generate *Atoh1-Brg1*^−/−^ mice. For timed pregnancies, the morning of plug was designated as embryonic day (E0.5) and the day of birth as postnatal day 0 (P0).

### Immunofluorescence staining and antibodies

Immunofluorescence staining was performed as described previously^61^. Briefly, cochleae were dissected and fixed in 4% paraformaldehyde in phosphate-buffered saline (PBS) at 4 °C overnight. Cochleae older than P5 were also decalcified in 10% EDTA. For sectioning, the samples were embedded in OCT compound and sectioned to an 8–10 μm thickness. For whole-mount immunostaining, the sensory epithelium was dissected and divided into apical, middle and basal parts. Samples were blocked for 30 min with 10% donkey serum, followed by incubation with primary antibodies in PBS at 4 °C overnight. After three washes with PBS, samples were incubated at 37 °C for 1 hour in secondary antibodies. F-actin filaments were visualized using rhodamine-labeled phalloidin, and DAPI was applied to stain nuclei. Images were acquired using a Leica LSM 700 laser scanning microscope or a Nikon TE2000 fluorescence microscope.

The following primary antibodies were used for immunostaining: anti-Brg1 (rabbit, 1:400, Abcam), anti-Myosin7a (rabbit, 1:400, Proteus Biosciences), anti-Myosin7a (goat, 1:800, Santa Cruz), anti-LGN (rabbit, 1:200, Proteintech), anti-Gαi3 (rabbit, 1:400, Sigma), anti-acetylated-α-tubulin (mouse, 1:400, Sigma), anti-α-tubulin (rabbit, 1:200, Proteintech), anti-βII-Spectrin (mouse, 1:400, BD), anti-Fz6 (goat, 1:500, RD), anti-Vangl1 (rabbit, 1:500, Sigma), anti-Prestin (goat, 1:400, Santa Cruz), anti-aPKC (rabbit, 1:200, Santa Cruz), anti-Cleaved-Caspase3 (rabbit, 1:400, CST), anti-ZO-1 (rabbit, 1:400, Invitrogen), anti-E-Cadherin (rabbit, 1:200, CST), anti-β-Catenin (rabbit, 1:400, Abcam), anti-Cyclin D1 (rabbit, 1:400, Abcam), anti-Ki67 (rabbit, 1:400, Abcam), anti-PCNA (rabbit, 1:400, Abcam), anti-Phospho-Histone3 (PH3) (rabbit, 1:800, Bioworld), anti-BrdU (mouse, 1:1000, Sigma), anti-Par3 (rabbit, 1:200, Proteintech).

### *In vitro* culture of the OC

OC samples were dissected on P5. The tissue pieces were mounted on poly-L-lysine-coated glass coverslips and cultured for 15 days in DMEM medium supplemented with 7% fetal bovine serum (Invitrogen) and 10 mg/ml ampicillin (Calbiochem) at 37 °C in 5% CO_2_ as previously described^62^.

### Scanning electron microscopy

Cochleae were dissected and fixed in 2.5% glutaraldehyde in PBS at 4 °C overnight. Cochleae were then dissected to expose the OC and post-fixed for 2 hours in 1% osmium tetroxide. Cochleae were dehydrated in a series of graded ethanol washes, critical point dried, mounted on metal stubs, and sputter coated with gold. Samples were imaged on aQUANTA FEG 250 scanning electron microscope at 5 kV.

### Transmission Electron Microscopy

Cochleae were dissected and fixed in 2.5% glutaraldehyde in PBS at 4 °C overnight. The sensory epithelium of the middle turn of the cochlear duct was dissected and post-fixed for 2 hours in 1% osmium tetroxide. Samples were embedded in Epon 812 resin after post-fixation, and ultra-thin sections (thickness, 70 nm) were cut on an ultramicrotome, placed on copper grids and examined on a JEOL-1200EX electron microscope at 80 kV.

### Auditory brainstem responses (ABR)

ABR measurements were performed as previously described^63^. In brief, mice were anesthetized with 0.007 g/ml pentobarbital sodium and were placed in a soundproof room. Needle electrodes were introduced just under the skin, with the active electrode placed between the ears above the vertex of the skull, the ground electrode between the eyes, and the reference electrode underneath the left ear. Mice were presented with click stimuli generated using a Tucker Davis Technologies(TDT) workstation running SigGen32 software(TDT). Auditory thresholds were determined by decreasing the sound intensities from 110 to 10 dB until the waveforms lost their reproducible morphology.

## Additional Information

**How to cite this article**: Jin, Y. *et al*. Deletion of Brg1 causes abnormal hair cell planer polarity, hair cell anchorage, and scar formation in mouse cochlea. *Sci. Rep.* **6**, 27124; doi: 10.1038/srep27124 (2016).

## Supplementary Material

Supplementary Information
