# Supplementary material for: Deletion of Brg1 causes abnormal hair cell planer polarity, hair cell anchorage, and scar formation in mouse cochlea
Source: Sci Rep. 2016 Jun 3;6:27124. doi: 10.1038/srep27124 (PMC4891731; doi:10.1038/srep27124)
Supplement: Supplementary Information [file srep27124-s1.pdf]

Deletion of Brg1 causes abnormal hair cell planer polarity, hair cell anchorage and scar formation in mouse cochlea

Yecheng Jin[1], Naixia Ren[1], Shiwei Li[1], Xiaolong Fu[1], Xiaoyang Sun[1], Yuqin Men[1], Zhigang Xu[1], Jian Zhang[1], Yue Xie[1], Ming Xia[2*] and Jiangang Gao[1*]

[1] School of Life Science and Key Laboratory of the Ministry of Education for Experimental Teratology, Shandong University, Jinan 250100, China.

[2] Department of Otolaryngology-Head and Neck Surgery, The Second Hospital of Shandong University, Jinan 250033, China.

*Correspondence and requests for materials should be addressed to M. X. (email: Xiamingsdu@sohu.com) or J.G. (email: jggao@sdu.edu.cn)

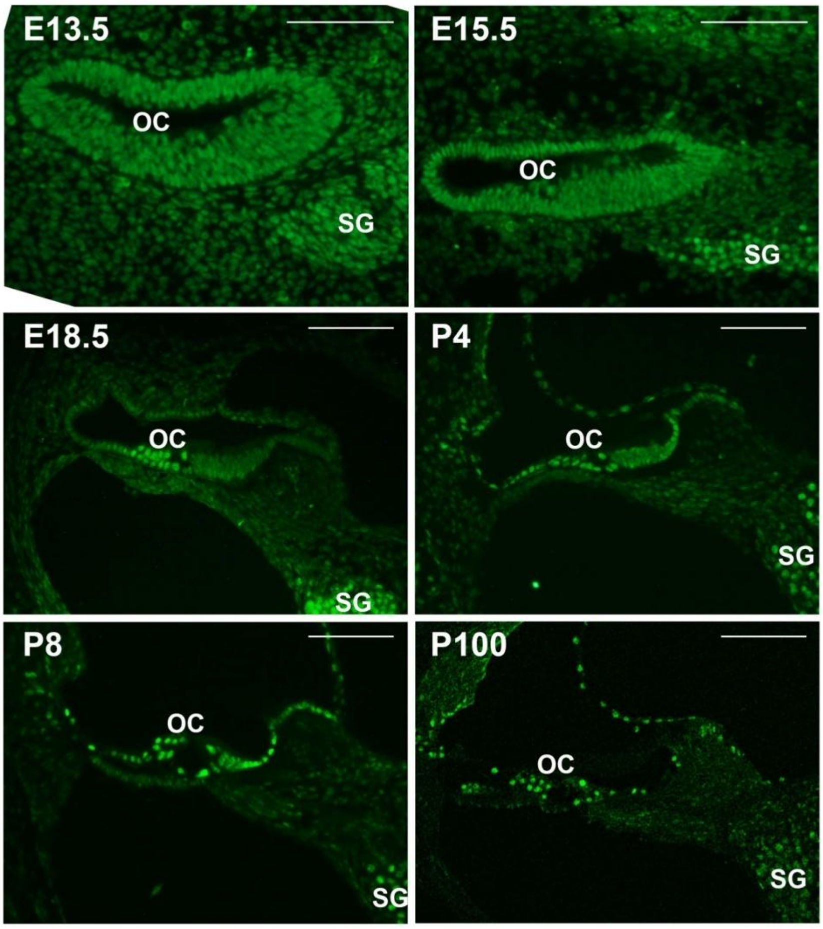

Supplementary Fig. S1. Brg1 expression in E13.5, E15.5, E18.5, P4, P8, and P100 cochlea. Transverse sections of E13.5, E15.5, E18.5, P4, P8, and P100 wild-type cochlea stained for Brg1 (green). Brg1 staining was stronger in HCs, SCs, and ganglion than other cell types. Abbreviations: OC, organ of Corti; SG, spiral ganglia. Scale bars: 100 μm.

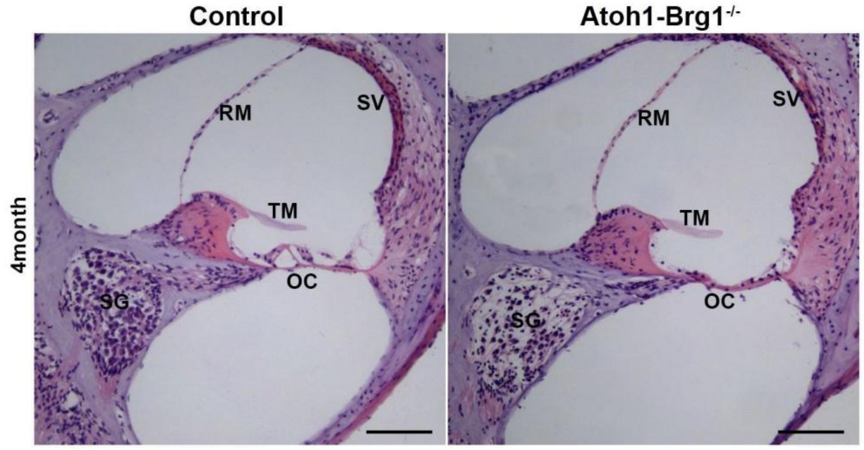

Supplementary Fig. S2. Degeneration of spiral ganglion neurons in *Atoh1-Brg1*$^{-/-}$ OC. Transverse sections of 4-month control and *Atoh1-Brg1*$^{-/-}$ cochlea stained with Hematoxylin and Eosin showed that HCs in the mutant mice were completely lost and the spiral ganglion neurons were reduced. Abbreviations: RM, Reissner's membrane; SV, stria vascularis; OC, organ of Corti; SG, spiral ganglia; TM, tectorial membrane. Scale bars: 100 µm.

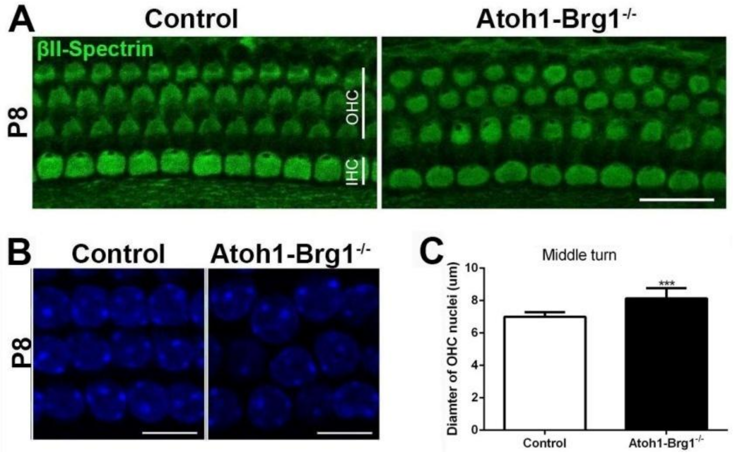

Supplementary Fig. S3. Abnormal remodeling of cuticular plate and nuclear morphology in Brg1-deficient HCs. (A)Whole-mount cochlea of P8 control and *Atoh1-Brg1*$^{-/-}$ mice stained with the cuticular plate marker βII-Spectrin revealed that the remodeling of the apical circumference was deficient in *Atoh1-Brg1*$^{-/-}$ HCs. OHC cuticular plates showed a non-convex shape with two lateral lobes flanking a membrane concavity on the OHC medial side and that the IHC cuticular plates had a

rounded rectangular shape in control mice at P8. In P8 *Atoh1-Brg1*$^{-/-}$ mice, lateral lobes and the negative curvature did not form at the OHC apical circumference, the cuticular plate was still a rounded hexagon shape, and IHC cuticular plate was an oval shape compared to rounded rectangular in the control mice. Scale bar: 20 µm. (B) OHC nuclei in P8 control and *Atoh1-Brg1*$^{-/-}$ whole-mount cochlea of middle turn were stained with DAPI (blue). The OHC nuclei in *Atoh1-Brg1*$^{-/-}$ cochlea were larger than the control OHC nuclei and sometimes showed an abnormal morphology. Scale bars: 10 µm. (C) Quantification of the OHC nuclei diameter at the middle turn of the P8 cochlea. The error bars indicate the SEM. ***P < 0.001 compared to the control by Student's t-test; n = 60 nuclei for each group.

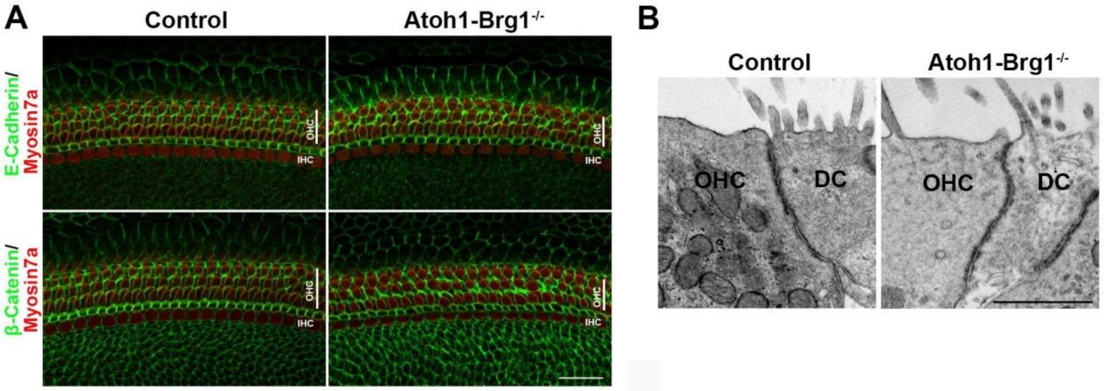

Supplementary Fig. S4. Apical tight junction was normal in *Atoh1-Brg1*$^{-/-}$ auditory epithelium. (A) The components of the special tight junction of auditory epithelium E-Cadherin and β-Catenin showed normal expression between the HC-SC and SC-SC apical junctions in *Atoh1-Brg1*$^{-/-}$ mice. The upper panel shows the whole-mount cochlea of P6 control and *Atoh1-Brg1*$^{-/-}$ mice stained with E-Cadherin (green) and the HC marker Myosin7a (red). The lower panel shows the whole-mount cochlea of P6 control and *Atoh1-Brg1*$^{-/-}$ mice stained with β-Catenin (green) and the OHC marker Myosin7a (red). Scale bar: 20 µm. (B) Transmission Electron Microscopy (TEM) images of the apical junction between HCs and SCs. The tight junction was not distinguishable between the *Atoh1-Brg1*$^{-/-}$ and control cochleae. Scale bar: 1 µm.

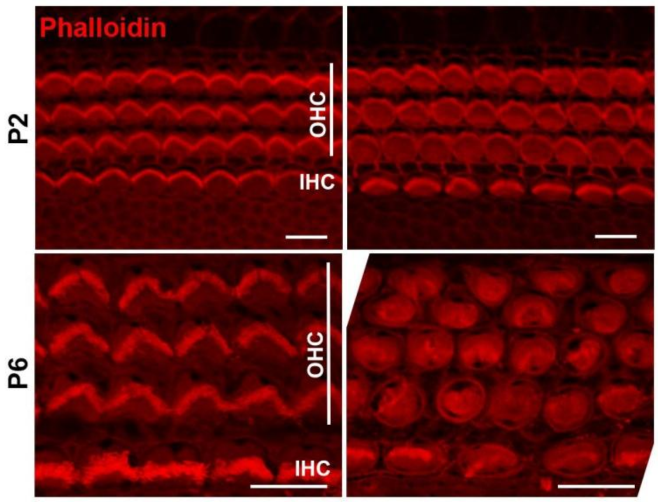

Supplementary Fig. S5. Stereocilia bundle defect of P2 and P6 *Atoh1-Brg1*$^{-/-}$ HC. (A) Confocal images of HCs in the middle-basal turn was stained with phalloidin. The stereocilia bundle defect was observed in P2 *Atoh1-Brg1*$^{-/-}$ cochlea. The stereocilia bundle became round shape in some OHC at P6 in *Atoh1-Brg1*$^{-/-}$ cochlea. Scale bars: 10 µm.

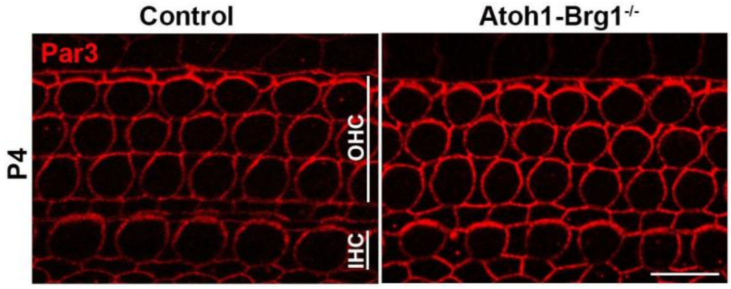

Supplementary Fig. S6. Par3 expression in P4 control and *Atoh1-Brg1*$^{-/-}$ auditory epithelium. Whole-mount cochlea of P4 control and *Atoh1-Brg1*$^{-/-}$ mice stained with Par3 (red). Par3 expressed in the apical junction of auditory epithelium both in control and mutant mice. Scale bar: 10 µm.

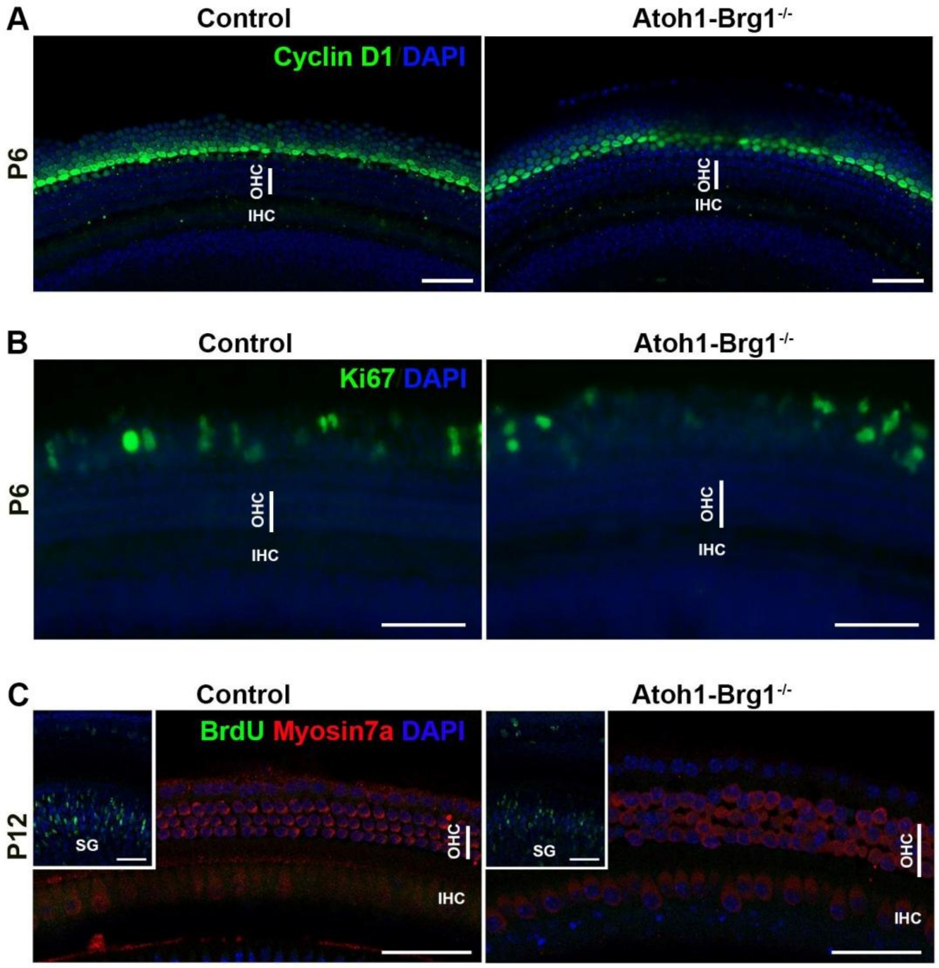

Supplementary Fig. S7. Brg1-deficient HCs were not stained by cell cycle markers. (A) Whole-mount cochlea of P6 control and *Atoh1-Brg1*$^{-/-}$ mice stained with Cyclin D1 (green) and DAPI (blue, nuclei). Scale bars: 50 μm. (B) Whole-mount cochlea of P6 control and *Atoh1-Brg1*$^{-/-}$ mice stained with Ki67 (green) and DAPI (blue, nuclei). Scale bars: 50 μm. (C) Whole-mount cochlea of P12 control and *Atoh1-Brg1*$^{-/-}$ mice stained with BrdU (green), HC marker Myosin7a (red), and DAPI (blue, nuclei). BrdU(50ug/g) was injected once a day from P6 to P10, then OC was dissected from P12 cochlea. Inserts show confocal Z-stack projections of neuron layer and act as positive control. Abbreviations: SG, spiral ganglia. Scale bars: 50 μm.
